# Migration of a Contraceptive Subdermal Device Into the Lung

**DOI:** 10.7759/cureus.48179

**Published:** 2023-11-02

**Authors:** Joud Enabi, Rami Al-Ayyubi, Pablo Amador, Alejandro Herrera, Devalla Deepika

**Affiliations:** 1 Internal Medicine, Texas Tech University Health Sciences Center, Odessa, USA; 2 Internal Medicine, Midland Memorial Hospital, Midland, USA

**Keywords:** lung, cardiology, foreign-body migration, drug implant, contraceptive implant

## Abstract

Subdermal contraceptive implants are usually inserted subdermally and carry the possibility to migrate within a small range, usually less than 2 cm from the insertion sites; significant migration over 2 cm is rare. This paper discusses the case of a 38-year-old female patient with a migrated subdermal Implanon contraceptive implant in the left pulmonary artery. On chest computed tomography, roughly a 4 cm long linear hyperdensity foreign body in the left lower lobe was found and was favored to be a migrated Implanon in a subsegmental pulmonary artery branch. An interventional radiologist performed an endovascular removal of the left pulmonary artery Implanon using a right common femoral vein access. Very few cases have been reported of complications with inserting and removing the subdermal contraceptive implants as it is considered a reasonably safe procedure in the hands of physicians familiar with the technique. Therefore, if a properly trained individual had carried out the correct procedure of inserting a subdermal implant, the migration of an implant over 2 cm should not occur.

## Introduction

Implanon is a 4 cm rod-shaped subdermal contraceptive implant coated with barium sulfate that is usually inserted into the inner side of the non-dominant arm. It is unusual for patients who undergo subdermal implant placement by adequately trained physicians to develop complications from the procedure. However, rare complications may include infections at the insertion site, injuries to nearby blood vessels and nerves, hematoma, or extensive fibrosis surrounding the implant [1]. Rarely subdermal contraceptive implants can migrate far from the insertion site [2]. As a result, a few cases in the literature have reported pulmonary embolization of the device [3]. In such cases, patients may be completely asymptomatic or present with symptoms such as dyspnea, hemoptysis, or chest pain. The implant is radiopaque and can be detectable by imaging techniques like X-ray or computed tomography (CT) scans. By the end of the use of contraceptive implants, extraction should be performed through a simple incision overlying the implant in the outpatient setting. In this case, we are reporting one of the rare cases of asymptomatic migration of Implanon to the pulmonary artery in a 38-year-old female from implantation to extraction. 

This article was previously presented as a meeting abstract at the 2023 Society of General Internal Medicine (SGIM) Annual Scientific Meeting in May 2023, in Colorado, United States.

## Case presentation

**Figure 1:**
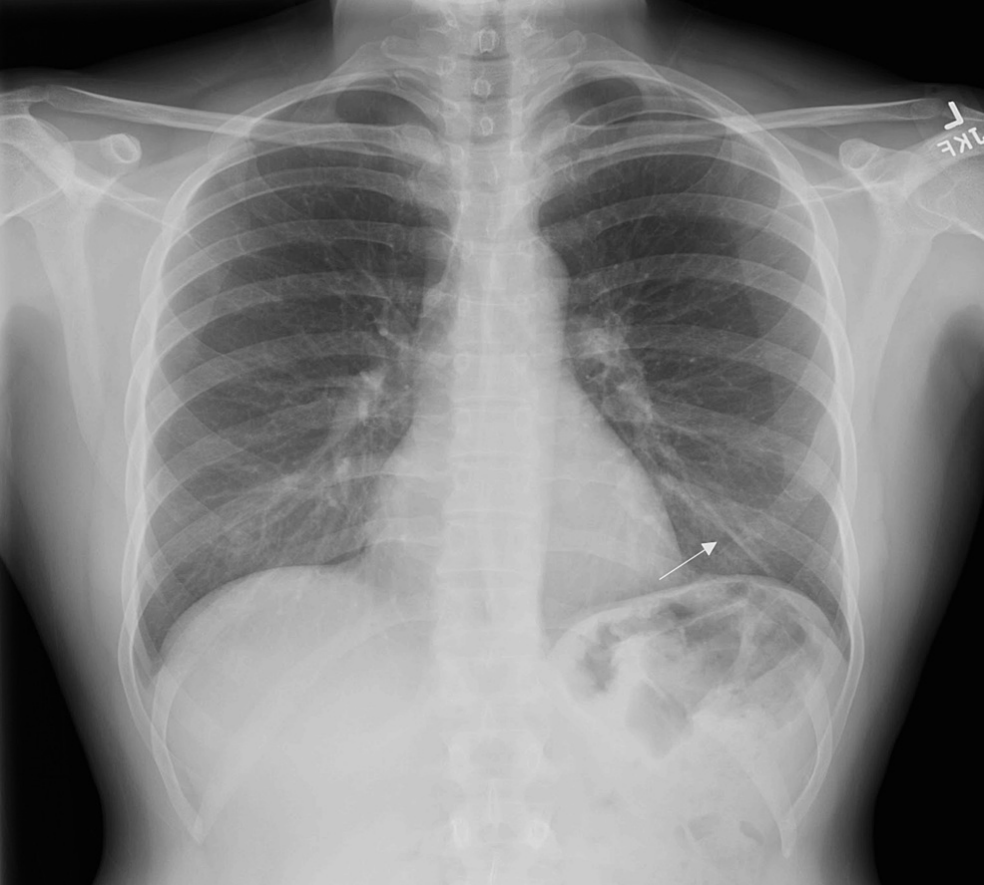
Chest X-ray showing implant in the left lung (arrow).

**Figure 2:**
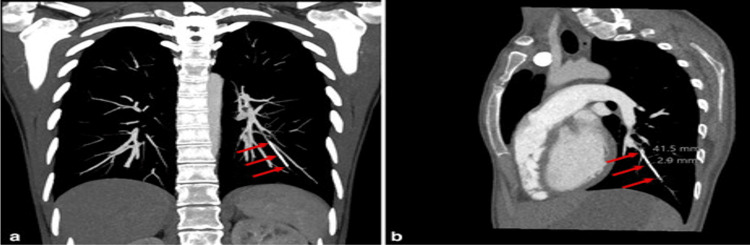
CT chest showing migrated implant into the left pulmonary artery branch (arrows).

A 38-year-old female with insignificant past medical/surgical histories had an Implanon subdermal contraceptive implant inserted in her left upper limb for contraception after delivering her third baby in June 2022. The patient experienced irregular vaginal spotting since the delivery. She was not able to feel the contraceptive implant after it was inserted and brought that up when she visited her gynecologist physician for a follow-up visit. An X-ray of the left arm was performed and it failed to detect the radiopaque Implanon; thus, she underwent a chest X-ray (Figure 1), and a linear foreign body projected in the region of the left lower lobe was found. The findings were highly suspicious for an embolized birth control device, and a CT scan of the chest was recommended for confirmation. On chest CT, a roughly 4 cm long linear hyperdensity foreign body in the left lower lobe was favored to be a migrated Implanon in the left subsegmental pulmonary artery branch (Figure 2). 

The patient denied any associated symptoms with the migrated implant, including chest pain, cough, hemoptysis, or dyspnea over the past three months. After consultations with interventional radiology, foreign body removal of the Implanon through an endovascular approach to the pulmonary artery using a right common femoral vein access was considered. The retrieval process was conducted with moderate sedation using the right common femoral vein as access. A triaxial system was employed, comprising a 12-F base sheath positioned at the level of the intrahepatic inferior vena cava (IVC), an 8-F curved sheath advanced to the proximal left pulmonary artery, and a 4-F angled diagnostic catheter in the left lower lobar artery. A 10-mm loop snare was utilized through the 4-F catheter to capture the upper part of the implant. While the device was successfully secured with the snare, it could not be drawn into the 8-F sheath because it had folded upon itself at the snare point. Out of concern for potential device damage with more forceful extraction, the snared device and inner sheath were carefully retracted under fluoroscopy through the right heart into the IVC. Subsequently, the system could be easily pulled into the 12-F sheath for complete retrieval. Following the removal of the device from the patient, its integrity was confirmed on the examination table. The patient was able to tolerate the procedure without complications and was discharged three hours later. She had a follow-up appointment with her gynecologist 40 days after the procedure and reported the complete resolution of her hormonal symptoms.

## Discussion

A subdermal contraceptive implant is a progesterone-only birth control method that contains 68 mg of etonogestrel [3]. Significant migration of a subdermal contraceptive implant over 2 cm is rare and occurs primarily caudally from the insertion site. The risk of other side effects is estimated to be roughly up to 1.1%[4]. These may include broken subdermal implants, fibrous adhesion formation around the implant, or deep insertion. In this case, it is estimated that during the insertion, the subdermal contraceptive implant was placed into the basilic vein. As a result, the subdermal birth-control implant migrated through the upper limb veins and settled in the pulmonary artery branch in the left posterior basal segment. Therefore, if a subdermal contraceptive implant is dislocated in the pulmonary artery, an intervention should be performed to remove it [5]. In this case, Interventional Radiology used the endovascular intervention approach to remove the left pulmonary artery Implanon.

Subdermal contraceptive implants should be placed in the groove between the biceps and triceps muscles at the inner side of the non-dominant arm above the elbow crease by 7 cm. The implant should be palpable throughout the use and can be detected on X-ray and CT imaging as it is coated with barium sulfate [6]. In this case, the patient was not able to feel the implant, and the physician recommended that she undergo an X-ray and CT imaging to localize the implant [7]. Studies show that the use of ultrasound is highly effective in removing impalpable implants when the subdermal implant is inserted deep into soft tissue [8]. The majority of impalpable contraceptive devices are removed using local anesthesia with the help of ultrasound. The ultrasound-guided dissection, using a 22-G spinal needle to stabilize the midpoint of the implant, is the most effective technique for removing such implants [9].

## Conclusions

Subdermal implants are generally safe and effective, but complications like migration can occur even when inserted by trained medical professionals. In our case, the implant migrated to the pulmonary artery through a basilic vein, highlighting the need for careful monitoring and prompt intervention. Fortunately, the patient didn't suffer any major consequences, and the migrated implant was successfully retrieved using endovascular techniques. Healthcare providers must educate patients about potential complications and emphasize the importance of making sure that the implant is palpable. Patients should be informed about reporting any concerns or unusual symptoms promptly. While complications with subdermal contraceptive implants are rare, healthcare professionals must remain alert and prepared to handle unexpected situations.
